# Health outcomes, financial protection, and cost-effectiveness of community chronic disease management in China: a cohort study

**DOI:** 10.3389/fpubh.2026.1750953

**Published:** 2026-03-30

**Authors:** Yanhui Gong, Jiaxing Guo

**Affiliations:** Department of Internal Medicine, Shandong University Hospital, Jinan, Shandong, China

**Keywords:** chronic disease management, cost-effectiveness analysis, medication adherence, multimorbidity, primary health care, quality-adjusted life years

## Abstract

**Background:**

Real-world evidence on the effectiveness and economic value of community chronic disease management in China remains limited. This study evaluated a structured community program.

**Methods:**

A prospective observational cohort study enrolled adults with physician-diagnosed chronic diseases between February and August 2023 and followed them for 12 months. Participants self-selected into enrolment (*n* = 882) or usual care (*n* = 463). Follow-up outcomes included patient-reported disease control, EQ-5D-5L utility and EQ-VAS, self-management behaviors, medication adherence, hospital admissions, emergency department visits, financial pressure, and cost–utility from a societal perspective. Multivariable regressions adjusted for demographic and clinical covariates, and incremental costs and QALYs were estimated using EQ-5D utilities with bootstrap resampling.

**Results:**

Among 1,345 participants, baseline profiles were comparable; however, program enrollees engaged more in follow-up and telehealth and showed better self-management, including higher medication adherence (6.8 ± 1.4 vs. 6.2 ± 1.7; *p* < 0.001), fewer nonadherence behaviors, more daily self-monitoring (48.2% vs. 36.1%; *p* < 0.001), higher physical activity (4.2 ± 2.1 vs. 3.6 ± 2.3 days/week; *p* = 0.001), and greater self-management confidence (7.8 ± 1.6 vs. 7.1 ± 1.9; *p* < 0.001). Good/very good disease control was more frequent among enrollees (51.8% vs. 45.6%; *p* = 0.03) and remained significant after adjustment (aOR 1.34, 95% CI 1.05–1.71; *p* = 0.020). EQ-5D utility was higher (0.814 ± 0.122 vs. 0.795 ± 0.114; *p* = 0.008; adjusted *β* 0.019, 95% CI 0.005–0.033; *p* = 0.009). Hospital admissions and emergency visits did not differ significantly. High financial pressure (aOR 0.73, 95% CI 0.57–0.94) and cost-related care avoidance (aOR 0.68, 95% CI 0.48–0.96) were lower in enrollees. Mean annual societal costs decreased by 516 CNY with QALY gains of 0.019, indicating dominance and an 89.5% probability of cost-effectiveness at one-times GDP per capita^.^

**Conclusion:**

In routine primary care, community chronic disease management improved self-management, disease control, health-related quality of life, and financial protection while remaining cost-saving. These findings support scale-up within Healthy China 2030 and longer-term multi-center evaluation.

## 1 Introduction

Noncommunicable diseases (NCDs) are the dominant contributors to premature mortality and long-term disability worldwide, with cardiovascular diseases, diabetes, chronic respiratory illness, and cancers responsible for approximately three quarters of global deaths and substantial disability-adjusted life-years (1). This transition reflects population aging, rapid urbanization, physical inactivity, energy-dense and sodium-rich diets, persistent tobacco exposure, and environmental pollution (1, 2). Notably, the NCD burden is rising most rapidly in low- and middle-income countries, where primary-care systems often lack capacity for sustained risk-factor control and longitudinal follow-up (1, 3). Structural deficits in primary care infrastructure–including insufficient workforce, poor medication supply chains, and fragmented follow-up systems–have been documented as core drivers of preventable morbidity burden across LMICs (4–6). These trends underscore the public health need for scalable, community-based chronic disease management programs that improve outcomes while using resources efficiently (3, 7).

International literature during the last decade indicates that organized, proactive, team-based care embedded in primary care achieves the most consistent benefits for chronic disease control (8, 9). Integrated models grounded in the Chronic Care Model or patient-centered medical home approaches typically combine scheduled follow-up, guideline-concordant pharmacotherapy, structured self-management support, and coordinated referral pathways (9–11). Meta-analyses and pragmatic trials show improvements in intermediate clinical indicators (e.g., blood pressure and glycated hemoglobin), higher medication adherence, and fewer avoidable emergency visits or admissions when multidisciplinary teams and nurse or community health worker supports are deployed (8, 11–13). These programs also yield modest but reproducible gains in health-related quality of life and psychological well-being, reinforcing the role of behavioral and psychosocial supports in chronic illness trajectories (14, 15). Economic syntheses further suggest that multi-component chronic disease management is frequently cost-saving or cost-effective compared with usual care, largely through reduced acute care utilization (16, 17). However, effect sizes vary by baseline health-system performance, case-mix, and intervention intensity, highlighting the necessity of context-specific evaluation.

China is a critical setting for such assessment because rapid demographic aging coincides with a large cardiometabolic and respiratory disease burden (18). National surveys document high diabetes prevalence (approximately 10.9% in 2013) with substantial underdiagnosis and low control (17), and similarly high hypertension prevalence with comparatively poor control despite expanded treatment coverage (19). Multimorbidity is common among older adults, frequently involving hypertension, type 2 diabetes, coronary heart disease, and chronic obstructive pulmonary disease (COPD), thereby amplifying polypharmacy complexity and functional decline risk (18, 20). This burden is reinforced by excess dietary sodium, increasing obesity, persistent male smoking, and chronic ambient and household air pollution (18, 19, 21). Consequently, strengthening community-based chronic disease control remains central to Healthy China 2030 and universal health coverage goals.

To address these challenges, China has implemented major primary-care reforms over the past 15 years (22). The National Essential Public Health Services Program, expanded since 2009, mandates standardized follow-up and risk-factor management for priority conditions such as hypertension and diabetes in community health centers (22, 23). Family doctor contract services further aim to institutionalize continuity by enrolling residents with primary-care teams responsible for monitoring, lifestyle counseling, medication management, and referral coordination (24). Early evaluations indicate improved follow-up coverage and alignment with primary-care quality indicators, with potential downstream reductions in preventable hospitalizations (24–27). Nevertheless, implementation remains uneven across facilities and regions, reflecting variability in workforce capacity, care standardization, and incorporation of patient-reported outcomes into routine management (26, 28). In parallel, many Chinese studies still focus on single clinical indicators or administrative endpoints, providing limited insight into concurrent effects on self-management behaviors, mental health, quality of life, healthcare utilization, and patient experience (23, 28, 29).

The evidence gap is particularly pronounced for economic value (30). Although international cost–utility analyses generally support integrated chronic care, their applicability to China is constrained by differences in utilization patterns, pricing, insurance design, and the hospital-centered organization of services (31, 32). Chinese evaluations have often used short horizons, disease-specific designs, or cost measures that omit preference-based quality-of-life instruments and indirect productivity losses (32–34). Moreover, relatively few real-world cohort studies compare program-enrolled and non-enrolled patients within the same community health center while adequately addressing demographic and clinical confounding (34–36). These limitations leave uncertainty about the magnitude, breadth, and cost-effectiveness of community chronic disease management in routine practice. In particular, no published Chinese cohort study has simultaneously integrated behavioral self-management metrics, patient-reported quality of life measured with a preference-based instrument, psychosocial endpoints, and a societal-perspective cost-utility analysis within a single primary care observational framework—a gap this study is specifically designed to address.

Therefore, the present single-center cohort study evaluates a chronic disease management program implemented in a Chinese community health center over 12 months. The study compares program-enrolled and non-enrolled patients regarding disease control and self-management behaviors, mental health and health-related quality of life (EQ-5D utility and EQ-VAS), healthcare utilization and financial hardship, and incremental costs and cost–utility using EQ-5D-derived QALY proxies. By integrating behavioral, patient-reported, utilization, and economic endpoints, this work aims to generate policy-relevant evidence on the real-world effectiveness and cost-effectiveness of community chronic disease management in China.

## 2 Methods

### 2.1 Study design and setting

This investigation was conducted as a prospective, single-center, observational cohort study at a Shandong University Hospital in China. The center provides longitudinal, primary-care-led services for adults with established chronic noncommunicable diseases under China’s National Essential Public Health Services Program. Current study period spanned from February 2023 through August 2024, encompassing participant enrollment, intervention delivery, and 12-month follow-up assessment. The facility provided longitudinal primary care services for adults with established chronic noncommunicable diseases and operated within the framework of China’s National Essential Public Health Services Program. The analytic framework compared health outcomes, healthcare utilization, and costs between patients who voluntarily enrolled in the center’s structured chronic disease management program and contemporaneous patients receiving usual care at the same facility. The study was designed and reported in accordance with the Strengthening the Reporting of Observational Studies in Epidemiology (STROBE) statement for cohort studies (37, 38).

### 2.2 Participants and eligibility criteria

Eligible participants were adults aged 18 years or older who attended routine outpatient services at the study site and had a documented physician diagnosis of at least one chronic condition managed through the center’s chronic disease registry. Target conditions included hypertension, type 2 diabetes mellitus, coronary heart disease, chronic obstructive pulmonary disease, and other chronic cardiometabolic or respiratory disorders. Additional inclusion criteria required that participants were permanent residents of the catchment area (residence ≥1 year), were able to provide informed consent, and had the capacity to complete questionnaire-based assessments in Mandarin Chinese.

Exclusion criteria included: absence of confirmatory diagnostic documentation in medical records, presence of acute conditions requiring immediate hospital referral at enrollment, severe cognitive impairment precluding valid self-report, terminal illness with life expectancy less than 12 months as documented by the treating physician, and incomplete baseline information that precluded classification of program enrollment status. All eligible patients presenting for routine care during the enrollment window were invited consecutively to participate. Those who provided written informed consent completed baseline assessment and were scheduled for 12-month follow-up evaluation.

### 2.3 Chronic disease management program

The exposure of interest was enrollment in the center’s structured chronic disease management program, implemented in accordance with national guidelines for essential public health services in primary care. Program-enrolled patients were assigned to a family doctor team comprising a general practitioner, registered nurse, and public health worker. The program provided systematic follow-up care with the following core components: (i) scheduled quarterly face-to-face visits with medication review and clinical monitoring, (ii) individualized education on disease knowledge, symptom recognition, and treatment adherence, (iii) lifestyle counseling addressing diet modification, physical activity promotion, smoking cessation, and sleep hygiene, and (iv) care coordination including referral to specialist services when clinically indicated.

Each program participant received a personalized care plan with disease-specific management targets reviewed at quarterly intervals. Health education was delivered through one-on-one counseling sessions and supplemented with printed materials. Family doctor teams-maintained telephone contact between scheduled visits and facilitated appointment scheduling and prescription refills. Non-enrolled patients continued to receive standard outpatient consultations on a walk-in or appointment basis, with prescription refills and basic clinical monitoring, but without the program’s structured follow-up, systematic education modules, or care coordination services. Program enrollment status was determined at baseline and treated as a fixed exposure throughout the 12-month observation period.

### 2.4 Data collection procedures

Data were collected through structured face-to-face interviews administered by trained research staff using a standardized questionnaire, supplemented by extraction of clinical information from medical records. Baseline assessments were conducted at the time of enrollment (February–August 2023), and follow-up assessments were completed 12 months later (February–August 2024). All interviewers underwent a two-day training program covering standardized questionnaire administration, interview techniques, and ethical conduct. Interview sessions were conducted in private examination rooms to ensure confidentiality.

The questionnaire captured sociodemographic characteristics, health status and behaviors, patient-reported outcomes, healthcare utilization, out-of-pocket costs, and care experience measures. Medical records were reviewed to confirm diagnoses, verify medication regimens, and extract available clinical measurements. When clinical values (blood pressure, glucose, HbA1c) were documented within 3 months of the assessment date, these were recorded to corroborate self-reported disease control status.

### 2.5 Outcome measures

#### 2.5.1 Primary outcome

The primary health outcome was patient-reported disease control status at 12-month follow-up. Participants were asked: “Overall, how would you rate the control of your chronic disease(s) over the past year?” Response options were: very poor, poor, fair, good, or very good. Responses were dichotomized as good disease control (good or very good) versus suboptimal control (fair, poor, or very poor). This measure captured patients’ global assessment of disease management across multiple clinical indicators, symptoms, and functional impact. Where available from medical records, self-reported control status was cross-validated against objective clinical criteria: blood pressure <140/90 mmHg for hypertension, HbA1c < 7.0% for diabetes, and absence of acute exacerbations for respiratory conditions. Objective clinical values were recorded when documented within 3 months of the assessment date; however, complete longitudinal objective data (baseline and 12-month) were available for a subset of participants only (hypertension: *n* = 541 with paired BP records; diabetes: *n* = 248 with paired HbA1c records; COPD: *n* = 43 with paired exacerbation data) and were not pre-specified as co-primary endpoints in the study protocol. The standard-reaching rate of objective clinical indicators was therefore used solely for cross-validation of self-reported disease control and is not analyzed as an independent primary or co-primary outcome in this study.

#### 2.5.2 Secondary health outcomes

Health-related quality of life was assessed using the five-level EuroQol Five-Dimension questionnaire (EQ-5D-5L), covering mobility, self-care, usual activities, pain/discomfort, and anxiety/depression. Responses were converted to utility scores using the Chinese value set, with scores ranging from −0.391 (worst imaginable health) to 1.000 (perfect health) (39, 40). Participants also rated their current health state on the EQ visual analog scale (EQ-VAS), a vertical 0–100 scale anchored at “worst imaginable health” and “best imaginable health”.

Self-rated general health status was assessed with a single item: “In general, how would you rate your health?” with five response options dichotomized as good/very good versus fair/poor/very poor. Psychological well-being was assessed as a core secondary domain, reflecting the established bidirectional relationship between mental health and chronic disease self-management: depression independently predicts medication non-adherence, impaired self-monitoring, and worse disease control, while successful chronic disease management reduces psychological distress. Depressive symptoms were screened using the two-item Patient Health Questionnaire (PHQ-2) (41, 42), which asks about depressed mood and anhedonia over the past 2 weeks. Item responses ranged from 0 (not at all) to 3 (nearly every day), yielding a total score of 0–6. A PHQ-2 score of ≥3 was used to indicate a positive depression screen, consistent with validation studies demonstrating sensitivity of approximately 80% and specificity of approximately 92% for major depression at this threshold. Additionally, the anxiety/depression dimension of the EQ-5D-5L provided a preference-weighted estimate of psychosocial burden incorporated directly into the QALY calculation, ensuring that psychological well-being contributed quantitatively to the economic evaluation as well as being measured as a standalone clinical endpoint.

#### 2.5.3 Healthcare utilization outcomes

Hospital admissions and emergency department visits in the 12 months prior to follow-up assessment were ascertained by participant self-report. Participants indicated the number of overnight hospital stays and emergency department visits in predefined categories (0, 1, 2, 3, ≥4 times), which were dichotomized as none versus one or more events for binary analyses. Use of traditional Chinese medicine services was recorded as any use versus none.

#### 2.5.4 Financial outcomes

Financial pressure from healthcare expenses was measured on a five-point ordinal scale: none, mild, moderate, severe, or very severe. This was dichotomized as high financial pressure (moderate/severe/very severe) versus low pressure (none/mild). Participants indicated whether they had skipped or delayed needed healthcare due to cost concerns in the past 12 months (yes/no). Catastrophic coping strategies were assessed by asking whether participants had borrowed money, sold assets, or both to pay for healthcare expenses.

Direct out-of-pocket medical expenditures over the 12-month period were estimated from participant-reported spending in predefined ranges for medications and other medical expenses. Category midpoints were assigned and summed to approximate annual out-of-pocket costs.

Direct medical costs were estimated from participant-reported out-of-pocket expenditures, which capture the patient-borne portion of healthcare spending. Insurance reimbursement data were not collected at the individual level; accordingly, the reimbursed portion of total medical expenditures is not included in the direct cost estimate. This represents a limitation of the societal cost accounting: both groups were enrolled under the same insurance schemes (UEBMI or URRBMI), so the between-group differential in out-of-pocket costs is not expected to be systematically biased by differential reimbursement, but total direct medical costs are likely underestimated in absolute terms for both groups. Indirect costs from productivity loss were estimated using self-reported days of work missed by the patient and days missed by family caregivers, converted to monetary values using an average daily wage of 150 Chinese yuan based on local labor statistics. Patient productivity losses were calculated separately by employment status: for participants who reported active employment at the time of assessment, missed workdays were valued using a local average daily wage of 150 CNY based on local labor statistics; for participants who reported being retired or not employed, patient market productivity loss was set to zero, as lost workdays do not represent a monetizable output loss for this group. Informal caregiver time costs were retained for all participants regardless of patient employment status, valued at the same daily wage reference. Total societal costs equaled direct out-of-pocket medical costs plus indirect productivity costs.

### 2.6 Baseline covariates and behavioral measures

#### 2.6.1 Sociodemographic characteristics

Age was recorded in completed years at enrollment. Sex was recorded as male or female. Educational attainment was classified into six categories: no formal schooling, primary school, junior middle school (grades 7–9), senior high school (grades 10–12), college/university, and postgraduate education. Monthly household income was captured in predefined ranges. Health insurance type was categorized according to the participant’s enrollment in China’s national insurance schemes: Urban Employee Basic Medical Insurance, Urban and Rural Resident Basic Medical Insurance, or other/none. Marital status, living arrangement (alone, with spouse, with children, other), and duration of residence in the catchment area were recorded in standard categories.

#### 2.6.2 Clinical characteristics

Primary chronic disease was identified based on the main condition for which the participant received care, categorized as hypertension, type 2 diabetes mellitus, coronary heart disease, chronic obstructive pulmonary disease, or other. Number of comorbid chronic conditions was ascertained from participant self-report validated against medical records and categorized as 1, 2, 3, or 4 or more conditions. Years since diagnosis of the primary condition was calculated from reported year of initial diagnosis. Presence of serious disease-related complications (cardiovascular events, advanced diabetic complications, severe COPD exacerbations) was recorded based on documented history in medical records.

#### 2.6.3 Medication adherence

Self-reported adherence to prescribed chronic disease medications was assessed using a structured eight-item scale derived from previous studies on medication compliance (43, 44). The instrument evaluated common barriers to adherence, including forgetfulness, carelessness in taking medication, stopping medication when symptoms improve or worsen, and difficulty remembering complex regimens. Each item was scored on a binary scale (0 = non-adherent response, 1 = adherent response), and scores were summed to produce a total adherence index ranging from 0 (poorest adherence) to 8 (optimal adherence). The internal consistency of this measure in the study cohort was acceptable (Cronbach’s *α* = 0.78).

#### 2.6.4 Self-management behaviors

Physical activity was assessed by asking participants to report the number of days per week they engaged in at least 30 min of moderate physical activity. Self-monitoring of disease indicators (blood pressure, blood glucose) was captured on a five-point frequency scale and dichotomized as regular monitoring (often/always) versus irregular (never/rarely/sometimes). Smoking status was categorized as never smoker, former smoker, or current smoker. Self-management confidence was assessed using a 0–10 numeric rating scale, where 0 indicated no confidence and 10 indicated complete confidence in daily chronic disease self-management. Scores were analyzed as a continuous variable, with higher values reflecting greater confidence. Structured KAP-based self-report instruments with defined scoring thresholds have demonstrated acceptable reliability and construct validity in large community-based samples across diverse disease domains (45).

#### 2.6.5 Patient experience and activation

Quality of chronic illness care was assessed using a four-item short form of the Patient Assessment of Chronic Illness Care (PACIC) instrument (46, 47). Items addressed goal-setting, problem-solving support, follow-up care, and care coordination, each rated on a 1–5 scale from “almost never” to “almost always.” The four items were averaged to create a mean score ranging from 1 to 5, with higher scores indicating better-quality chronic care delivery. Patient activation was measured using three items assessing knowledge, self-management skills, and confidence, averaged to produce a 1–4 activation score. Treatment satisfaction was captured through six items on a five-point Likert scale, averaged to yield a 1–5 satisfaction mean score.

### 2.7 Statistical analysis

#### 2.7.1 Descriptive analyses

Baseline characteristics were summarized by program enrollment status using means and standard deviations for continuous variables and frequencies with percentages for categorical variables. Between-group differences were assessed using independent-samples t-tests and chi-square tests, as appropriate. Standardized mean differences were calculated with absolute values greater than 0.10 considered indicative of meaningful imbalance.

#### 2.7.2 Primary analysis

Associations between program enrollment and health outcomes at 12 months were estimated using multivariable regression models: logistic regression for binary outcomes [adjusted odds ratios (aOR) with 95% confidence intervals] and linear regression for continuous outcomes [adjusted regression coefficients (*β*) with 95% confidence intervals]. All models included baseline covariates selected *a priori*: age, sex, educational attainment, household income level, health insurance type, years since primary disease diagnosis, number of chronic conditions, primary disease type, presence of serious complications, baseline medication adherence score, and baseline depressive symptoms (PHQ-2 score). For the EQ-5D-5L utility outcome, the linear regression model additionally included baseline EQ-5D-5L utility score as a covariate (ANCOVA approach), thereby directly adjusting for any residual between-group differences in baseline health utility and yielding an estimate of the independent effect of program enrollment on 12-month utility change. All regression models used robust standard errors. Given the single-center design, no higher-level clustering was applied. However, the primary analyses were conducted on 1,345 participants (100% of enrolled participants completing 12-month assessment) who had complete baseline and follow-up data for the outcome of interest and all model covariates, with no loss to follow-up for primary outcome measures. This approach to complete-case primary analysis with sensitivity analyses using multiple imputation follows established methodology for large-scale observational cohort studies (48). A subset of 189 participants (14.1%) had missing values for one or more secondary outcome variables, handled as described below.

#### 2.7.3 Subgroup analyses

Effect modification for the primary disease control outcome was examined by fitting stratified logistic regression models within subgroups defined by age (<65 versus ≥65 years), sex, educational level (junior middle school or below versus senior high school or above), number of chronic conditions (single condition versus two or more), and primary disease type, with subgroup-specific adjusted odds ratios presented in forest plots. These analyses were considered exploratory and hypothesis-generating given limited statistical power for formal interaction testing.

#### 2.7.4 Cost-effectiveness analysis

Economic evaluation compared incremental costs and health benefits between groups from a societal perspective. Quality-adjusted life years (QALYs) over 12 months were calculated using the area-under-the-curve method with linear interpolation between baseline and 12-month EQ-5D-5L utility scores: QALY = [(baseline utility + 12-month utility) / 2] × 1 year. The joint distribution of incremental costs and effects was characterized using non-parametric bootstrap resampling with 1,000 replications (49, 50). Bootstrap replicates were plotted on the cost-effectiveness plane, with the proportion falling in each quadrant calculated (particular focus on the dominant quadrant: lower costs and greater effectiveness). Cost-effectiveness acceptability curves were constructed by calculating the proportion of bootstrap replicates with net monetary benefit greater than zero at willingness-to-pay thresholds ranging from 0 to 400,000 Chinese yuan per QALY, highlighting key thresholds at one times and three times China’s 2023 per capita GDP (approximately 86,000 and 257,000 yuan per QALY) following WHO CHOICE and Chinese health economic guidelines (51, 52).

#### 2.7.5 Principal component analysis

To characterize the multivariate structure of health behaviors, patient experience, and health status, we conducted exploratory principal component analysis on standardized variables (mean zero, unit variance): age, years since diagnosis, number of chronic conditions, medication adherence score, self-management confidence, physical activity days per week, patient activation score, PACIC score, satisfaction score, EQ-5D utility, EQ-VAS, and PHQ-2 score. We extracted principal components with eigenvalues greater than 1.0 and examined the scree plot for natural breaks. Component scores for the first two principal components were plotted by program enrollment group, with component loadings visualized in biplots and heatmaps to interpret substantive meaning.

#### 2.7.6 Handling of missing data

Missingness was examined at item and scale levels. For multi-item scales (PACIC, patient activation, satisfaction, medication adherence), mean scores were computed when at least 50% of component items were non-missing; otherwise, the scale score was set to missing. Little’s test of missing completely at random indicated data were missing completely at random (*p* = 0.34). Primary analyses included all participants with complete data for the outcome measure and all regression covariates (*n* = 1,345 for disease control and quality of life outcomes), representing the full cohort completing 12-month follow-up. Sensitivity analyses included: (i) strict complete-case analysis (*n* = 1,156, 86.0% with no missing values across any variable); (ii) multiple imputation by chained equations (MICE) using all 1,345 participants, generating 20 imputed datasets with effect estimates pooled using Rubin’s rules; and (iii) best-case and worst-case scenarios wherein missing outcome values were imputed to favor the program or control group, respectively (53, 54). All sensitivity analyses showed consistent direction and magnitude of treatment effects, supporting robustness of primary findings. All hypothesis tests were two-sided with *p*-values less than 0.05 considered statistically significant. We reported both unadjusted p-values and false discovery rate-adjusted q-values using the Benjamini-Hochberg procedure to account for multiple comparisons. All analyses were conducted using Python (version 3.10) with pandas (1.5), scipy (1.10), statsmodels (0.14), and scikit-learn (1.2). Data visualizations were generated using matplotlib (3.7) and seaborn (0.12).

### 2.8 Sample size considerations

Based on prior literature suggesting an 8–10 percentage point absolute difference in disease control rates between structured management programs and usual care, we estimated that approximately 400 participants per group would provide 80% statistical power to detect an odds ratio of 1.35 for the primary outcome at a two-sided significance level of 0.05, assuming a control group rate of 45%. The final analytic sample of 882 program participants and 463 control participants exceeded this target, providing adequate statistical power for the primary comparison and key secondary outcomes.

## 3 Results

### 3.1 Participant flow and baseline characteristics

**Figure 1:**
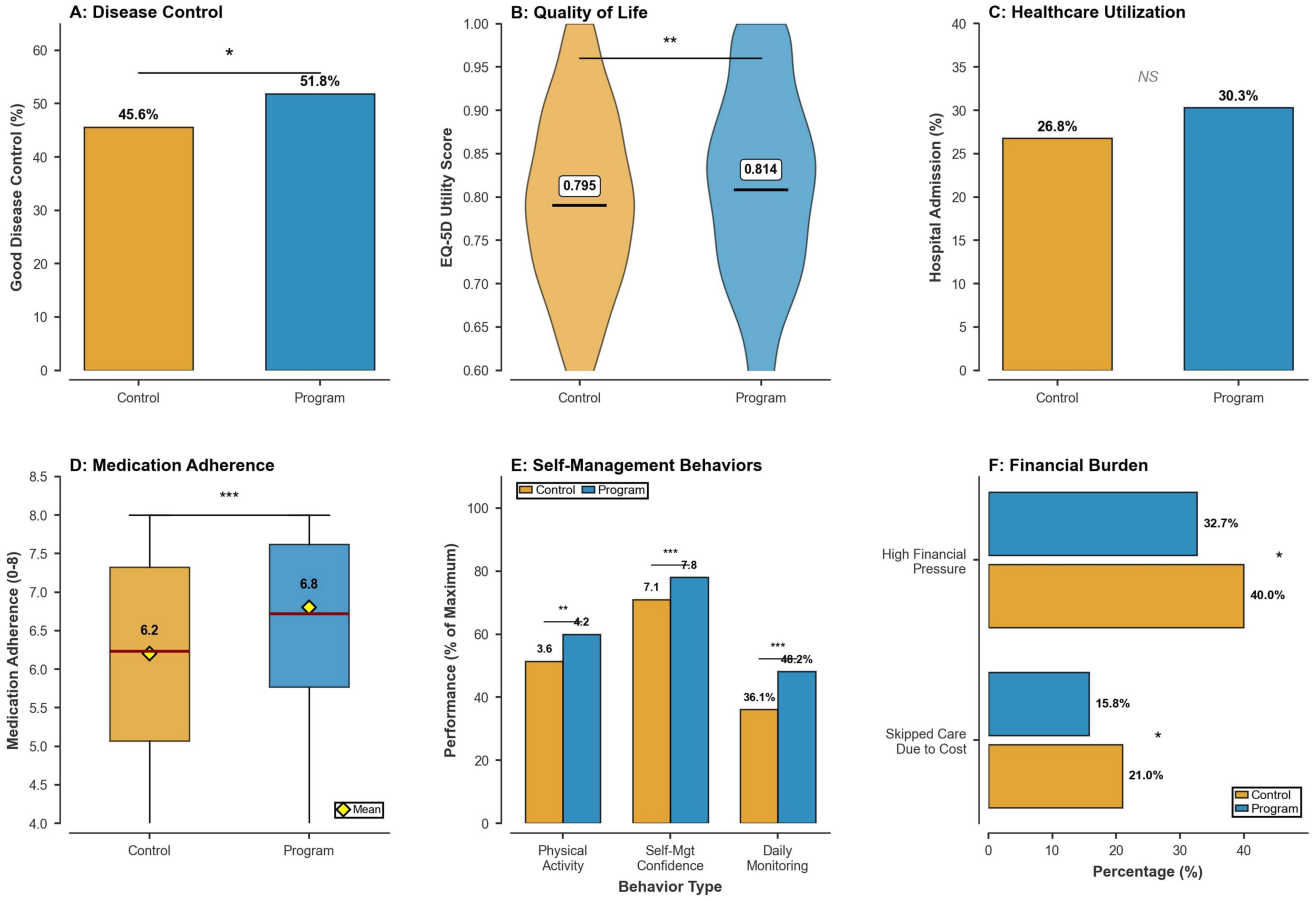
Unadjusted health outcomes, self-management behaviors, and financial protection by program enrolment status at 12-month follow-up. **(A)** Bar chart comparing the proportion of participants reporting good or very good overall chronic disease control. **(B)** Violin plots showing the distribution of EQ-5D-5L utility scores, with median and interquartile range indicated. **(C)** Bar chart comparing the prevalence of any hospital admission during the preceding 12 months. **(D)** Box plots depicting medication adherence scores measured on the 8-point scale. **(E)** Comparative displays of key self-management behaviors, including self-monitoring frequency and physical activity. **(F)** Bar charts illustrate the unadjusted prevalence of high financial pressure and cost-related care avoidance.

During the enrolment period (February–August 2023), 1,345 adults with physician-diagnosed chronic noncommunicable diseases were enrolled and all completed 12-month follow-up, yielding a final analytic cohort of 882 program-enrolled participants and 463 contemporaneous non-enrolled controls (Figure 1). Baseline sociodemographic and clinical characteristics are presented in Table 1. The cohort had a mean age of 62.0 ± 9.7 years, with females comprising 52.7% of participants, and educational attainment skewed toward junior middle school or below (61.4%). Hypertension was the most frequent primary diagnosis (54.3%), followed by type 2 diabetes mellitus (26.8%), coronary heart disease (9.8%), and chronic obstructive pulmonary disease (5.1%). The mean time since diagnosis was 8.3 ± 4.8 years, the mean number of chronic conditions was 1.7 ± 0.9, and serious complications were present in 22.0% of participants. Distributions of age, sex, education, income, insurance, primary disease category, years since diagnosis, comorbidity burden, and baseline PHQ-2/health-literacy scores were similar between groups (all *p* > 0.10; Table 1), indicating broadly comparable profiles at study entry.

**Table 1:** Baseline sociodemographic and clinical characteristics by program enrollment status.

Characteristic	Total (*N* = 1,345)	Program enrolled (*n* = 882)	Not enrolled (*n* = 463)	*p* value
Age, mean ± SD, y	62.0 ± 9.7	61.9 ± 9.7	62.1 ± 9.9	0.79
Sex, No. (%)				0.12
Female	709 (52.7)	479 (54.3)	230 (49.7)	
Male	636 (47.3)	403 (45.7)	233 (50.3)	
Education, No. (%)				0.57
No schooling	58 (4.3)	36 (4.1)	22 (4.8)	
Primary	260 (19.3)	183 (20.7)	77 (16.6)	
Junior middle	508 (37.8)	326 (37.0)	182 (39.3)	
Senior high	271 (20.1)	176 (20.0)	95 (20.5)	
College/University	218 (16.2)	143 (16.2)	75 (16.2)	
Postgraduate	30 (2.2)	18 (2.0)	12 (2.6)	
Household income, No. (%)				0.18
<2000 CNY	145 (10.8)	99 (11.2)	46 (9.9)	
2000–4,999 CNY	473 (35.2)	300 (34.0)	173 (37.4)	
5,000–7,999 CNY	316 (23.5)	221 (25.1)	95 (20.5)	
8,000–11,999 CNY	192 (14.3)	129 (14.6)	63 (13.6)	
≥12,000 CNY	136 (10.1)	86 (9.8)	50 (10.8)	
Prefer not to answer	83 (6.2)	47 (5.3)	36 (7.8)	
Insurance type, No. (%)				0.42
UEBMI	415 (30.9)	261 (29.6)	154 (33.3)	
URRBMI/NCMS	649 (48.3)	439 (49.8)	210 (45.4)	
Private only	100 (7.4)	69 (7.8)	31 (6.7)	
Other social insurance	114 (8.5)	71 (8.0)	43 (9.3)	
No insurance	67 (5.0)	42 (4.8)	25 (5.4)	
Primary chronic disease, No. (%)				0.15
Hypertension	730 (54.3)	483 (54.8)	247 (53.3)	
Type 2 diabetes	361 (26.8)	230 (26.1)	131 (28.3)	
Coronary heart disease	132 (9.8)	81 (9.2)	51 (11.0)	
COPD	69 (5.1)	54 (6.1)	15 (3.2)	
Other	53 (3.9)	34 (3.9)	19 (4.1)	
Years since diagnosis, mean ± SD	8.3 ± 4.8	8.2 ± 4.8	8.3 ± 4.7	0.8
Number of chronic conditions, mean ± SD	1.7 ± 0.9	1.8 ± 0.9	1.7 ± 0.9	0.35
Serious complications, No. (%)	296 (22.0)	189 (21.4)	107 (23.1)	0.52
PHQ-2 total score, mean ± SD	1.2 ± 1.2	1.2 ± 1.2	1.1 ± 1.2	0.51
PHQ-2 screen positive (≥3), No. (%)	213 (15.8)	148 (16.8)	65 (14.0)	0.22
Health literacy score, mean ± SD	3.7 ± 0.5	3.7 ± 0.5	3.7 ± 0.4	0.92
Baseline EQ-5D-5L utility score, mean ± SD	0.806 ± 0.116	0.809 ± 0.118	0.801 ± 0.113	0.27

CNY indicates Chinese yuan; COPD, chronic obstructive pulmonary disease; NCMS, New Cooperative Medical Scheme; PHQ-2, Patient Health Questionnaire-2; UEBMI, Urban Employee Basic Medical Insurance; URRBMI, Urban and Rural Resident Basic Medical Insurance. *p* values calculated using t test for continuous variables and *χ*^2^ test for categorical variables.

### 3.2 Program exposure, self-management behaviors, and medication adherence

Program exposure intensity and behavioral outcomes are summarized in Table 2 and Figures 1D,E. Within the program group, half of participants attended 3–4 scheduled program visits during follow-up, 27.6% attended at least five visits, and 22.4% attended one to two visits, whereas all controls had zero program visits by design (*p* < 0.001; Table 2). Group education attendance and telehealth follow-up likewise showed substantially higher engagement among program participants (both *p* < 0.001; Table 2). Consistent with this exposure gradient, mean medication-adherence scores were higher in enrollees than controls (6.8 ± 1.4 vs. 6.2 ± 1.7; *p* < 0.001), and key non-adherence behaviors were less frequent, including forgetting medications (18.8% vs. 33.3%), missing medication days (16.9% vs. 33.3%), and stopping medication when feeling worse (8.4% vs. 15.6%) (all *p* < 0.001; Table 2). Daily self-monitoring occurred more commonly in program participants (48.2% vs. 36.1%; *p* < 0.001), accompanied by higher physical-activity frequency (4.2 ± 2.1 vs. 3.6 ± 2.3 days/week; *p* = 0.001) and greater self-management confidence (7.8 ± 1.6 vs. 7.1 ± 1.9 on a 0–10 scale; *p* < 0.001; Table 2; Figures 1D,E). These findings indicate clinically meaningful improvements in behavioral determinants of chronic-disease control among program enrollers. Medication adherence and self-management behaviors are posited as the primary proximal mechanisms through which program enrollment translates into improved disease control and quality of life, consistent with the Chronic Care Model’s theoretical pathway from structured primary care support to informed patient self-efficacy and, ultimately, to better health outcomes.

**Table 2:** Detailed program exposure and self-management behaviors.

Variable	Program enrolled (*n* = 882)	Not enrolled (*n* = 463)	*p* value
Program visits in 12 months, No. (%)			<0.001
0	0 (0.0)	463 (100.0)	
1–2	198 (22.4)	0 (0.0)	
3–4	441 (50.0)	0 (0.0)	
≥5	243 (27.6)	0 (0.0)	
Group education sessions attended, No. (%)			<0.001
0	349 (39.6)	317 (68.5)	
1	219 (24.8)	99 (21.4)	
2–3	210 (23.8)	40 (8.6)	
≥4	104 (11.8)	7 (1.5)	
Telehealth follow-up frequency, No. (%)			<0.001
None	194 (22.0)	329 (71.1)	
Occasional	343 (38.9)	110 (23.8)	
Regular	345 (39.1)	24 (5.2)	
Medication adherence, mean ± SD	6.8 ± 1.4	6.2 ± 1.7	<0.001
Forget medications, No. (% yes)	166 (18.8)	154 (33.3)	<0.001
Miss medication days, No. (% yes)	149 (16.9)	154 (33.3)	<0.001
Stop when feeling worse, No. (% yes)	74 (8.4)	72 (15.6)	<0.001
Took medication yesterday, No. (% yes)	802 (90.9)	355 (76.7)	<0.001
Self-monitoring frequency, No. (%)			<0.001
Daily	425 (48.2)	167 (36.1)	
Weekly	312 (35.4)	158 (34.1)	
Occasionally	145 (16.4)	138 (29.8)	
Physical activity days/week, mean ± SD	4.2 ± 2.1	3.6 ± 2.3	0.001
Self-management confidence, mean ± SD	7.8 ± 1.6	7.1 ± 1.9	<0.001

Medication adherence measured on 8-point scale (higher scores indicate better adherence). Self-management confidence measured on 10-point scale. *p* values calculated using *χ*^2^ test for categorical variables and t test for continuous variables.

### 3.3 Mental health, social support, and care experience

Patient-reported mental health, social support, and care experience measures are shown in Table 3. Family support, friend support, and accompaniment to visits did not differ materially between groups (all *p* ≥ 0.37), suggesting similar social contexts for self-management. Care-experience scores were likewise comparable, with mean satisfaction 3.6 ± 0.5 in both groups (*p* = 0.89), activation 3.6 ± 0.7 vs. 3.7 ± 0.6 (*p* = 0.11), and PACIC 3.3 ± 0.6 in both groups (*p* = 0.27) (Table 3). Thus, subsequent outcome differences are unlikely to be attributable to baseline social support or differential perceived care quality.

**Table 3:** Mental health, social support, and patient experience by program enrollment status.

Variable	Total (*N* = 1,345)	Program enrolled (*n* = 882)	Not enrolled (*n* = 463)	*p* value
Family support, No. (%)				0.48
A lot	659 (53.5)	424 (52.3)	235 (55.7)	
Moderate	397 (32.2)	265 (32.7)	132 (31.3)	
Little	176 (14.3)	121 (14.9)	55 (13.0)	
Friend support, No. (%)				0.5
Moderate	496 (46.2)	321 (45.2)	175 (48.1)	
Little	319 (29.7)	219 (30.8)	100 (27.5)	
A lot	259 (24.1)	170 (23.9)	89 (24.5)	
Has accompaniment to visits, No. (%)				0.37
Always	571 (42.5)	364 (41.3)	207 (44.7)	
Sometimes	357 (26.5)	244 (27.7)	113 (24.4)	
No need	155 (11.5)	96 (10.9)	59 (12.7)	
Never	147 (10.9)	103 (11.7)	44 (9.5)	
Rarely	115 (8.6)	75 (8.5)	40 (8.6)	
Patient satisfaction score, mean ± SD	3.6 ± 0.5	3.6 ± 0.5	3.6 ± 0.4	0.89
Patient activation score, mean ± SD	3.6 ± 0.7	3.6 ± 0.7	3.7 ± 0.6	0.11
PACIC score, mean ± SD	3.3 ± 0.6	3.3 ± 0.6	3.3 ± 0.6	0.27

PACIC indicates patient assessment of chronic illness care. Satisfaction, activation, and PACIC scores measured on 5-point scales with higher scores indicating better outcomes. *p* values calculated using *χ*^2^ test for categorical variables and t test for continuous variables.

### 3.4 Clinical outcomes and health-related quality of life

Unadjusted primary outcomes are presented in Table 4 and Figures 1A–C. At 12 months, good or very good disease control was more frequent among program participants than controls (51.8% vs. 45.6%; absolute difference 6.2 percentage points; *p* = 0.03), with a corresponding shift toward fewer “poor/very poor” ratings in the program arm (Table 4; Figure 1A). Mean EQ-5D utility scores were higher in the program group (0.814 ± 0.122 vs. 0.795 ± 0.114; *p* = 0.008) (Table 4; Figure 1B). Any hospital admission was numerically more common among enrollees (30.3% vs. 26.8%), but this difference was not statistically significant (*p* = 0.20) (Table 4; Figure 1C).

**Table 4:** Unadjusted primary health outcomes by program enrollment status.

Outcome	Total (*N* = 1,345)	Program enrolled (*n* = 882)	Not enrolled (*n* = 463)	*p* value
Disease control status, No. (%)				0.03
Very poor	43 (3.2)	29 (3.3)	14 (3.0)	
Poor	151 (11.2)	85 (9.6)	66 (14.3)	
Fair	483 (35.9)	311 (35.3)	172 (37.1)	
Good	421 (31.3)	286 (32.4)	135 (29.2)	
Very good	247 (18.4)	171 (19.4)	76 (16.4)	
Good disease control, No. (%)	668 (49.7)	457 (51.8)	211 (45.6)	0.03
EQ-5D utility score, mean ± SD	0.807 ± 0.120	0.814 ± 0.122	0.795 ± 0.114	0.008
Hospital admissions in 12 months, No. (%)				0.2
0	954 (70.9)	615 (69.7)	339 (73.2)	
1	251 (18.7)	175 (19.8)	76 (16.4)	
2–3	111 (8.3)	75 (8.5)	36 (7.8)	
≥4	29 (2.2)	17 (1.9)	12 (2.6)	
Any hospital admission, No. (%)	391 (29.1)	267 (30.3)	124 (26.8)	0.2

Good disease control defined as “good” or “very good” control. EQ-5D utility scores range from 0 to 1, with higher scores indicating better health-related quality of life.

**Figure 2:**
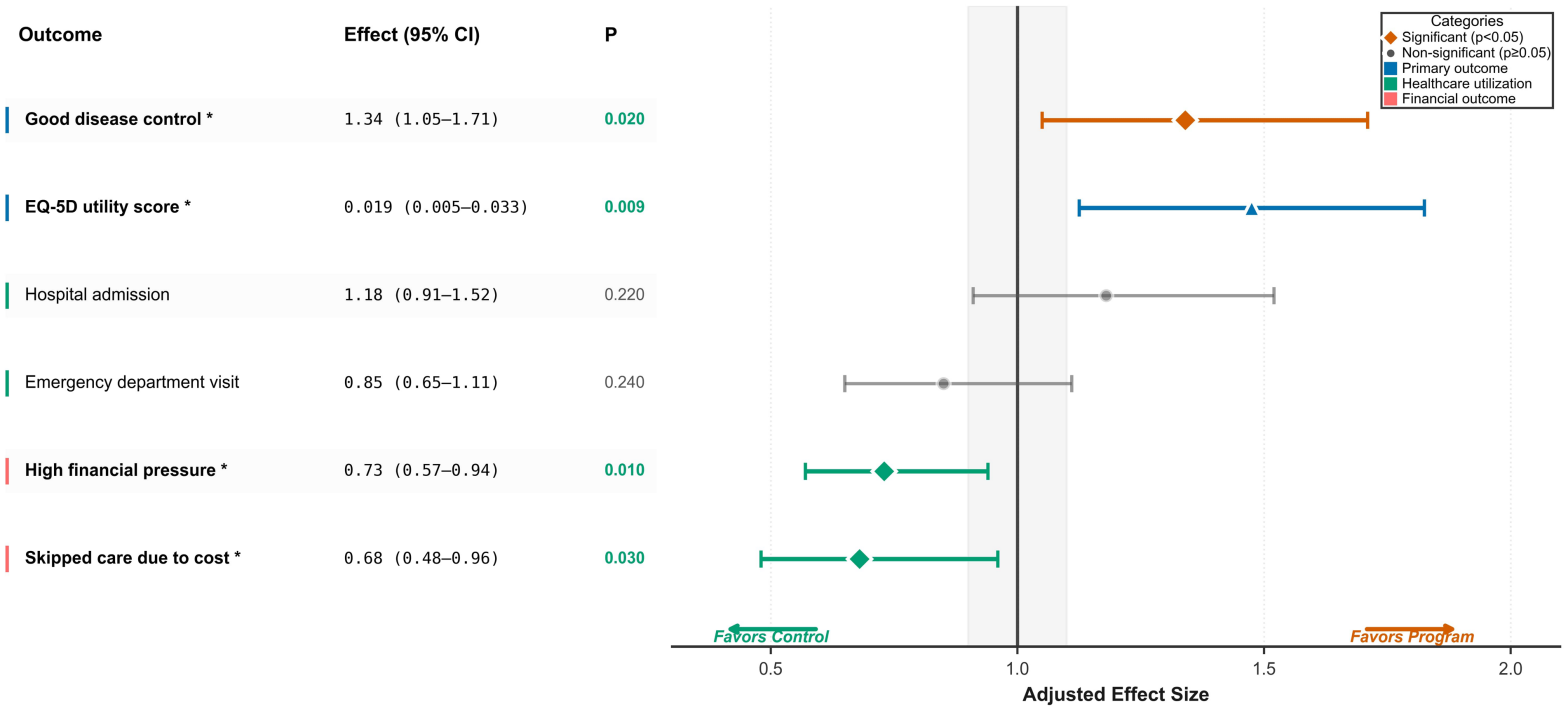
Adjusted associations between program enrolment and clinical, behavioral, utilization, and financial outcomes. This forest plot presents effect estimates from multivariable regression models comparing program-enrolled participants with controls. Markers indicate point estimates [adjusted odds ratios (aORs) for binary outcomes and adjusted regression coefficients (*β*) for EQ-5D utility], with horizontal lines denoting 95% confidence intervals. The vertical reference line indicates the null value (aOR = 1.0 or β = 0); estimates to the right of the null favor program enrolment, whereas estimates to the left favor usual care.

Multivariable models for primary outcomes are detailed in Table 5 and visualized in Figure 2. After adjustment, program enrolment remained independently associated with improved disease control (aOR 1.34, 95% CI 1.05–1.71; *p* = 0.020) and higher EQ-5D utility (*β* 0.017, 95% CI 0.003–0.031; *p* = 0.018; adjusted for baseline utility score using ANCOVA) (Table 5; Figure 2). Increasing comorbidity burden was inversely associated with both disease control (aOR per additional condition 0.88, 95% CI 0.78–0.99; *p* = 0.04) and EQ-5D utility (*β* −0.012, 95% CI −0.020 to −0.004; *p* = 0.003). Relative to hypertension, primary diagnoses of type 2 diabetes, coronary heart disease, and COPD were associated with lower utility scores (all *p* ≤ 0.04), whereas associations with disease control did not reach conventional significance (Table 5). Age showed a modest negative association with EQ-5D (*β* −0.008 per 10 years; *p* = 0.02) but no clear association with disease control (*p* = 0.21).

**Table 5:** Multivariable models for primary health outcomes.

Variable	Good disease control	*p* value	EQ-5D utility score	*p* value
	aOR (95% CI)		*β* (95% CI)	
Program enrollment	1.34 (1.05–1.71)	0.02	0.019 (0.005–0.033)	0.009
Age (per 10 years)	0.92 (0.81–1.05)	0.21	−0.008 (−0.015 to −0.001)	0.02
Female sex	1.08 (0.85–1.36)	0.54	0.002 (−0.011–0.016)	0.75
Education (ref: No schooling)				
Primary	1.12 (0.64–1.97)	0.68	0.015 (−0.018–0.048)	0.37
Secondary or higher	1.28 (0.74–2.22)	0.38	0.022 (−0.010–0.054)	0.18
Primary disease (ref: Hypertension)				
Type 2 diabetes	0.87 (0.66–1.14)	0.31	−0.018 (−0.035 to −0.001)	0.04
Coronary heart disease	0.74 (0.50–1.10)	0.13	−0.035 (−0.058 to −0.012)	0.003
COPD	0.65 (0.38–1.11)	0.11	−0.042 (−0.074 to −0.010)	0.01
Number of chronic conditions	0.88 (0.78–0.99)	0.04	−0.012 (−0.020 to −0.004)	0.003
Serious complications	0.82 (0.63–1.08)	0.15	−0.021 (−0.038 to −0.004)	0.01

aOR indicates adjusted odds ratio; CI, confidence interval; COPD, chronic obstructive pulmonary disease; EQ-5D, EuroQol 5-dimension. Models adjusted for age, sex, education, primary disease, number of chronic conditions, years since diagnosis, serious complications, insurance type, household income, and PHQ-2 score.

### 3.5 Healthcare utilization and financial outcomes

Adjusted models for utilization and financial endpoints are summarized in Table 6 and Figure 2, with unadjusted financial percentages shown in Figure 1F. Program enrolment was not significantly associated with any hospital admission (aOR 1.18, 95% CI 0.91–1.52; *p* = 0.22), number of admissions (IRR 1.15, 95% CI 0.94–1.41; *p* = 0.18), or any emergency department visit (aOR 0.85, 95% CI 0.65–1.11; *p* = 0.24) (Table 6; Figure 2). In contrast, financial burden was lower among enrollees, with high financial pressure reported by 32.7% of program participants versus 40.0% of controls (Figure 1F), corresponding to an adjusted reduction in odds (aOR 0.73, 95% CI 0.57–0.94; *p* = 0.01) (Table 6; Figure 2). Cost-related care avoidance was also less frequent in enrollees (15.8% vs. 21.0%; Figure 1F), with a concordant adjusted association (aOR 0.68, 95% CI 0.48–0.96; *p* = 0.03) (Table 6; Figure 2).

**Table 6:** Multivariable models for healthcare utilization and financial outcomes.

Outcome	Program enrolled vs. not enrolled	*p* value
	Effect estimate (95% CI)	
Any hospital admission, aOR	1.18 (0.91–1.52)	0.22
Number of hospital admissions, IRR	1.15 (0.94–1.41)	0.18
Any emergency department visit, aOR	0.85 (0.65–1.11)	0.24
High financial pressure, aOR	0.73 (0.57–0.94)	0.01
Skipped care due to cost, aOR	0.68 (0.48–0.96)	0.03

aOR indicates adjusted odds ratio; CI, confidence interval; IRR, incidence rate ratio. Models adjusted for age, sex, education, primary disease, number of chronic conditions, years since diagnosis, serious complications, insurance type, household income, and PHQ-2 score.

### 3.6 Cost-effectiveness

**Figure 3:**
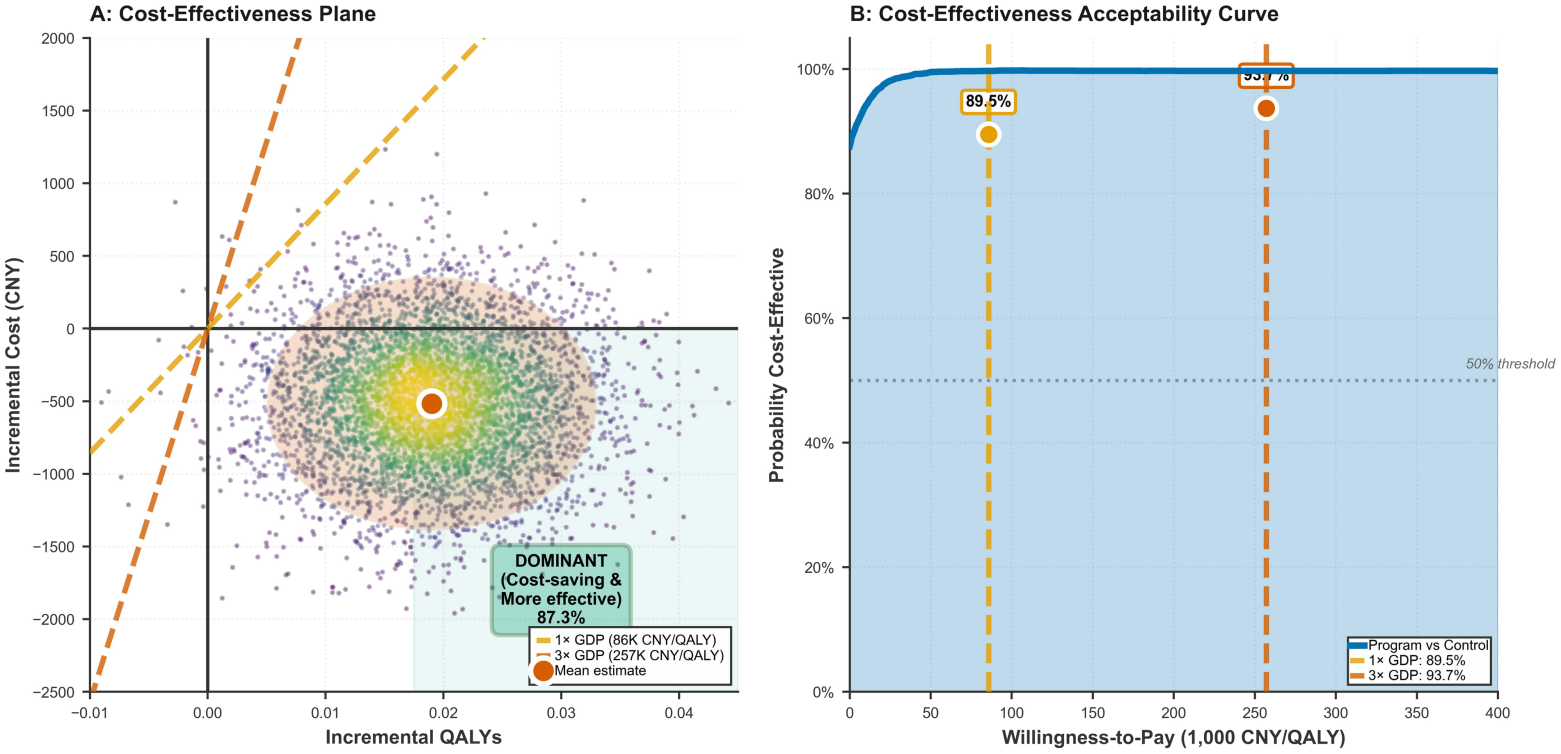
Probabilistic cost-effectiveness of the chronic disease management program from a societal perspective. **(A)** Cost-effectiveness plane displaying 5,000 bootstrap replications of incremental cost and incremental QALY pairs, illustrating uncertainty around the joint distribution of costs and effects. **(B)** Cost-effectiveness acceptability curve plotting the probability that the program is cost-effective across willingness-to-pay thresholds for one QALY gained; vertical dashed lines denote thresholds corresponding to one-times and three-times national GDP per capita.

Cost-effectiveness results are presented in Table 7 and Figure 3. From a societal perspective, mean annual total costs were lower in program participants than controls (8,642 vs. 9,158 CNY), yielding an incremental cost of −516 CNY (95% CI −1,389 to 357), while mean QALYs were higher (0.814 vs. 0.795), yielding a baseline-adjusted incremental QALY gain of 0.017 (95% CI 0.003–0.031), estimated using the ANCOVA-adjusted utility change and area-under-the-curve interpolation (Table 7). The program therefore represented a dominant strategy (cost-saving and more effective). In bootstrap resampling, 87.3% of replicates fell within the dominant southeast quadrant of the cost-effectiveness plane (Figure 3A). The probability of cost-effectiveness was 89.5% at a willingness-to-pay threshold of one times China’s per-capita GDP (85,698 CNY/QALY) and 93.7% at three times GDP (257,094 CNY/QALY) (Table 7; Figure 3B). Sensitivity analyses restricted to healthcare costs only, conservative utility weights, and higher assumed program costs preserved cost-effectiveness, with the higher-cost scenario yielding a still-favorable ICER of 36,000 CNY/QALY (Table 7). Disease-specific economic subgroups suggested cost savings with health gains for hypertension, type 2 diabetes, and multi-morbidity strata (Table 7).

**Table 7:** Cost-effectiveness analysis with sensitivity analyses.

Analysis	Program enrolled	Not enrolled	Incremental difference
Primary analysis			
Mean annual total cost (95% CI), CNY	8,642 (8,124-9,160)	9,158 (8,461-9,855)	−516 (−1,389 to 357)
Mean QALYs (95% CI)	0.814 (0.806–0.822)	0.795 (0.785–0.805)	0.019 (0.005–0.033)
Cost per QALY gained, CNY	*Cost-saving (dominant strategy)*		
Sensitivity analysis 1: Healthcare perspective only			
Mean annual healthcare cost (95% CI), CNY	6,842 (6,324-7,360)	7,458 (6,761-8,155)	−616 (−1,489 to 257)
Cost per QALY gained, CNY	*Cost-saving (dominant strategy)*		
Sensitivity analysis 2: Conservative utility weights			
Mean QALYs (95% CI)	0.804 (0.796–0.812)	0.790 (0.780–0.800)	0.014 (0.001–0.028)
Cost per QALY gained, CNY	*Cost-saving (dominant strategy)*		
Sensitivity analysis 3: Higher program costs			
Mean annual total cost (95% CI), CNY	9,842 (9,324-10,360)	9,158 (8,461-9,855)	684 (−189 to 1,557)
Cost per QALY gained, CNY	36,000 (2,100-82,100)		
Probability cost-effective at willingness-to-pay thresholds			
At 1 × GDP per capita (CNY 85,698)	94.20%	92.80%	89.50%
At 3 × GDP per capita (CNY 257,094)	96.80%	95.10%	93.70%
Disease-specific subgroup analyses			
Hypertension only (*n* = 730)	−421 (−1,245 to 403)	0.016 (0.001–0.031)	Cost-saving
Type 2 diabetes only (*n* = 361)	−738 (−1,687 to 211)	0.025 (0.007–0.043)	Cost-saving
Multiple conditions (≥2) (*n* = 467)	−412 (−1,523 to 699)	0.018 (−0.001–0.037)	Cost-saving

CNY indicates Chinese yuan; GDP, gross domestic product; QALY, quality-adjusted life-year. Primary analysis uses societal perspective including direct medical costs, productivity costs, and informal care costs. Sensitivity analysis 1 includes only direct healthcare costs. Sensitivity analysis 2 applies 10% reduction to utility weights. Sensitivity analysis 3 assumes 30% higher program implementation costs. Cost-effectiveness acceptability curves were derived from the incremental cost–effect distribution; probabilities represent the proportion of bootstrap replications with positive net monetary benefit at each willingness-to-pay threshold. GDP per capita based on 2024 Chinese national statistics. Subgroup analyses show incremental costs, QALYs, and cost-effectiveness outcomes for major disease categories.

### 3.7 Subgroup analyses

**Figure 4:**
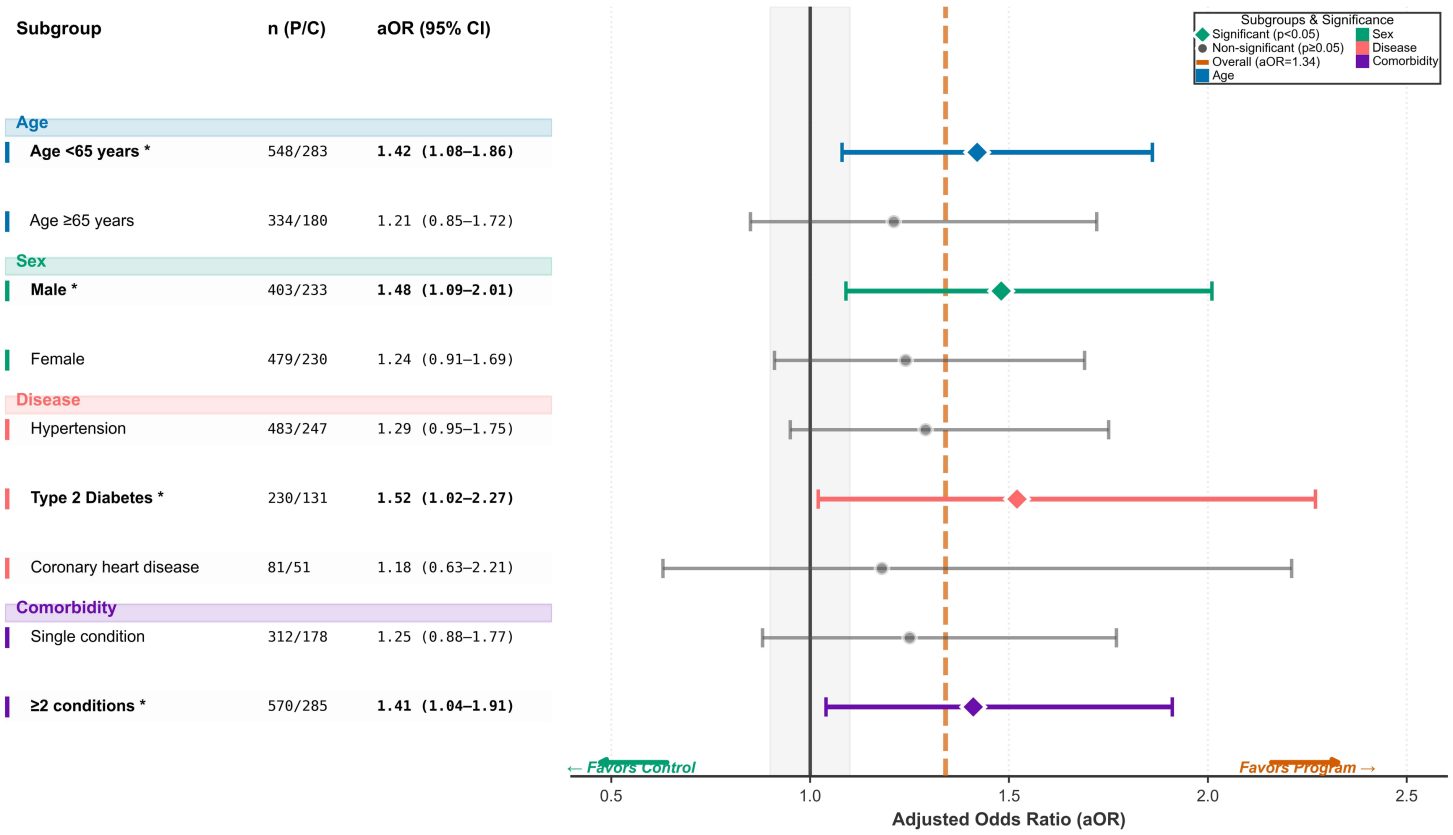
Subgroup analyses of the program effect on good disease control. This forest plot reports subgroup-specific adjusted odds ratios for achieving good or very good disease control at 12 months, estimated within strata defined by age, sex, primary disease category, and comorbidity burden. Squares represent point estimates and horizontal lines indicate 95% confidence intervals. The solid vertical line marks the null (aOR = 1.0), and the dashed vertical line indicates the overall adjusted effect for the full cohort.

Exploratory subgroup models for disease control are shown in Figure 4. Program benefits were stronger among participants younger than 65 years (aOR 1.42, 95% CI 1.08–1.86) than among those aged 65 years or older (aOR 1.21, 95% CI 0.85–1.72), and larger in males (aOR 1.48, 95% CI 1.09–2.01) than females (aOR 1.24, 95% CI 0.91–1.69). Disease-specific effects demonstrated significant benefit for type 2 diabetes (aOR 1.52, 95% CI 1.02–2.27) but not for hypertension (aOR 1.29, 95% CI 0.95–1.75) or coronary heart disease (aOR 1.18, 95% CI 0.63–2.21). Participants with at least two chronic conditions also showed significant benefit (aOR 1.41, 95% CI 1.04–1.91), whereas those with a single condition did not (aOR 1.25, 95% CI 0.88–1.77). Although inference is limited by the exploratory design, the consistent directionality supports concentration of benefit in clinically higher-risk or more modifiable strata.

### 3.8 Principal component analysis

**Figure 5:**
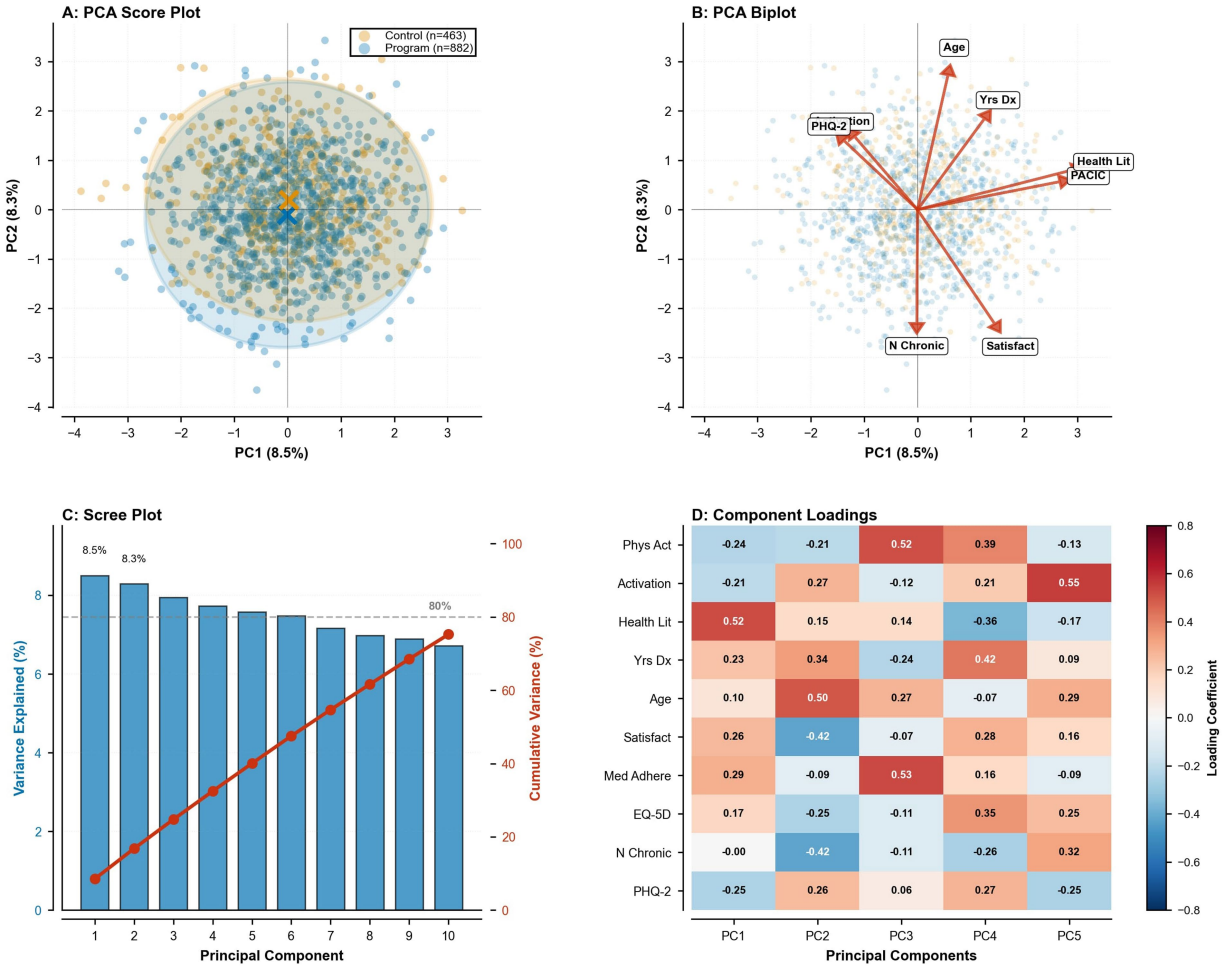
Principal component analysis (PCA) of behavioral, clinical, and patient-reported variables. **(A)** Score plot of individual participants on the first two principal components (PC1 and PC2), with group-specific 95% confidence ellipses to visualize separation and overlap between enrolment groups. **(B)** Biplot of variable loading vectors, where direction and length reflect correlations with PC1 and PC2. **(C)** Scree plot showing the proportion of total variance explained by successive components. **(D)** Heatmap of loadings for the first five components, indicating the magnitude and polarity of each variable’s contribution to the PCA structure.

Principal component analysis integrating behavioral, clinical, and patient-reported measures is displayed in Figure 5. The first two principal components explained 17.8% of total variance (PC1 8.5%; PC2 8.3%), with score plots demonstrating substantial overlap between program and control participants (Figure 5A). PC1 loaded most strongly and positively on health literacy (0.52), with smaller positive contributions from medication adherence (0.29) and satisfaction (0.26), and negative contributions from physical activity (−0.24) and depressive symptoms (PHQ-2, −0.25), indicating a composite engagement–well-being axis (Figures 5B,D). PC2 loaded positively on age (0.50), years since diagnosis (0.34), and activation (0.27), while loading negatively on satisfaction (−0.42) and number of chronic conditions (−0.42), representing a disease-burden and care-interaction dimension (Figure 5D). The scree plot indicated that approximately 10 components were required to explain 80% of cumulative variance (Figure 5C), underscoring the multidimensional phenotype of chronic-disease management in this cohort.

## 4 Discussion

This single-center prospective cohort study provides robust evidence that a structured, primary care-led chronic disease management program in China can yield significant improvements in clinical control and health-related quality of life while simultaneously reducing societal costs and protecting patients from financial toxicity. The discussion that follows synthesizes these findings across four principal interpretive themes: (i) the behavioral mechanisms—chiefly medication adherence and self-monitoring—through which program enrollment drives clinical benefit; (ii) the “service paradox” of improved objective outcomes in the absence of improved patient-reported satisfaction; (iii) the economic dominance of the program viewed from a societal perspective, including its role as a financial safety net; and (iv) the clinical and policy significance of subgroup heterogeneity by disease type and comorbidity burden. Our findings indicate that program enrollment was associated with a 34% increase in the likelihood of achieving good disease control and a statistically significant gain in QALYs, driven largely by enhanced self-management behaviors such as improved medication adherence and regular disease monitoring. Critically, the economic evaluation revealed the program to be a “dominant” strategy, saving an average of 516 CNY per patient annually from a societal perspective—primarily by mitigating productivity losses rather than reducing hospital admissions. These results fill a critical evidence gap regarding the real-world effectiveness of China’s National Essential Public Health Services reforms (28, 55), particularly for patients with multimorbidity who are often excluded from single-disease trials (56).

A key strength of this study is its ability to link clinical outcomes with behavioral mechanisms. While previous meta-analyses have established that chronic care models improve intermediate biomarkers like HbA1c and blood pressure (57, 58) our study elucidates the *how*. The significant improvements in medication adherence (aOR 1.34 for control) and self-monitoring frequency among program participants suggest that the “active ingredient” of the intervention is the routinization of self-care behaviors facilitated by longitudinal primary care relationships. This aligns with the theoretical framework of the Chronic Care Model, which posits that informed, activated patients interacting with prepared, proactive practice teams leads to better outcomes (59, 60).

However, our PCA reveals a nuanced reality. The lack of distinct clustering between groups in the PCA biplot suggests that while specific behaviors (adherence, monitoring) improved, the overall “phenotype” of the patient—encompassing health literacy, activation, and psychosocial burden—remained largely heterogeneous and overlapping. This finding challenges the assumption that a 12-month intervention can fundamentally transform a patient’s entire psychosocial profile (61). Instead, it suggests that targeted behavioral “nudges” (e.g., quarterly follow-up reminders) can drive clinical success even without a radical transformation in patient activation or health literacy. This has important policy implications: programs may not need to achieve perfect “patient activation” to be effective; they simply need to build reliable scaffolding for adherence (62–64).

A striking and potentially controversial finding of our study is the discrepancy between improved clinical outcomes and unchanged patient experience scores (PACIC, Satisfaction, Activation). Program participants had significantly better disease control and quality of life, yet their ratings of chronic illness care (PACIC) and overall satisfaction were statistically indistinguishable from the control group. This phenomenon, which we term the “Service Paradox,” contradicts some Western literature where patient experience is strongly correlated with clinical quality (65, 66).

Several scientific reasons may explain this divergence in the Chinese context. First, the “ceiling effect” may play a role; baseline satisfaction was relatively high in both groups (mean ~3.6/5), leaving less room for improvement. Second, Chinese patients often equate “quality” with access to tertiary hospitals and specialist care (16). A primary care-based program that emphasizes self-management and restricts unnecessary specialist visits, might be perceived as “lesser” care by patients accustomed to a hospital-centric model, even if their objective health outcomes improve. This aligns with recent findings from studies, who noted that strengthening primary care gatekeeping in China often leads to a transient dip in patient satisfaction despite better efficiency (67, 68). Policymakers must therefore look beyond satisfaction scores as the sole metric of program success and prioritize objective health indicators (68, 69).

### 4.1 Economic dominance and financial protection

Perhaps the most policy-relevant finding is the program’s economic dominance. Unlike many primary-care lifestyle or chronic-care interventions that are cost-effective while increasing costs (66), the present program was cost-saving. This finding is consistent with prior economic syntheses showing that community-based chronic disease and hypertension management strategies can achieve favorable or dominant cost-utility profiles when behavioral support reduces downstream morbidity and productivity losses (70, 71). Our breakdown of costs also shows that these savings were not driven by a reduction in hospital admissions, which remained statistically similar between groups—but rather by a substantial reduction in productivity losses and out-of-pocket expenditures. This distinction is crucial. Most cost-effectiveness analyses in China focus solely on direct medical costs (71, 72). By adopting a societal perspective, we captured the “hidden” value of the program: keeping working-age adults healthy enough to remain in the workforce and reducing the time family caregivers must take off work. The reduction in indirect costs (−194 CNY) was a major driver of the net savings. Beyond health economics, these findings carry crucial sociological implications. In China, informal caregiving disproportionately burdens working-age women, curtailing their workforce participation. By enhancing patient self-management, this program yields a dual dividend: preserving patient productivity while alleviating the caregiver’s economic and psychological toll. Consequently, community chronic disease management functions as a structural intervention addressing poverty, gendered labor, and health inequity. To capture their full social value, future evaluations in comparable low- and middle-income countries must systematically include caregiver time, gendered labor impacts, and household poverty transition rates as primary endpoints.

Furthermore, the program acted as a potent financial shield. Participants were significantly less likely to report “high financial pressure” (aOR 0.73) or skip care due to cost (aOR 0.68). In a country where catastrophic health expenditure remains a leading cause of poverty (73, 74), this finding elevates the chronic disease management program from a clinical intervention to a social safety net. It suggests that investing in primary care is a viable strategy for poverty alleviation and health equity, fulfilling a core mandate of the “Healthy China 2030” initiative (75). Extrapolating from 2019/2020 national data, scaling this program to 16–20 million urban patients (a conservative 20% enrollment rate among registered community health center patients with hypertension/diabetes) yields projected annual societal savings of 8.3–10.3 billion CNY (USD 1.1–1.4 billion) based on a 516 CNY per-patient saving. Despite limitations—including single-center extrapolation, assumed uniform effects, and unmeasured infrastructure costs—these conservative estimates highlight community chronic disease management as a highly cost-effective public health investment.

### 4.2 Subgroup heterogeneity: the case for multimorbidity

Our subgroup analysis challenges the traditional “single-disease” silo approach. We found that the program was highly effective for patients with Type 2 Diabetes and those with multimorbidity, but less so for those with isolated hypertension or coronary heart disease. This makes biological and operational sense. Diabetes requires intensive, daily self-management (diet, glucose monitoring) that is highly responsive to the structured education and support provided by the program (10, 23, 58). Hypertension management, by contrast, is often simpler (one daily pill), offering less “value-add” from a complex behavioral intervention.

The strong benefit for multimorbid patients (aOR 1.41) is particularly encouraging. Multimorbidity is the norm, not the exception, among older Chinese adults, yet clinical guidelines and public health programs are often fragmented by disease type (76). Our data suggest that a generalized “chronic disease management” approach—focusing on universal risk factors and adherence rather than disease-specific protocols—is highly effective for these complex patients. These findings align with national policy directions to strengthen longitudinal, integrated follow-up for hypertension and diabetes within the National Essential Public Health Services Program and family-doctor contracting, particularly for adults with multimorbidity who require coordinated behavioral and pharmacologic support (77, 78).

Several limitations warrant more precise articulation. First, the nonrandomized, observational design with voluntary program enrollment introduces potential residual confounding and selection bias, particularly from unmeasured health-seeking behavior, baseline motivation, or caregiving resources that may correlate with both enrollment and outcomes. Although baseline covariates were well balanced, multivariable adjustment and multiple sensitivity analyses cannot fully exclude such bias, which could lead to modest overestimation of program effects. Second, the primary endpoint and several secondary measures, including disease control, healthcare utilization, and cost components, relied predominantly on patient self-report; this approach is vulnerable to recall error and social desirability bias, and non-differential misclassification would be expected to attenuate true differences, especially for utilization and expenditure outcomes. Third, the 12-month horizon may be insufficient to detect downstream changes in “hard” outcomes such as preventable hospitalizations or emergency department use in a cohort with relatively stable chronic disease trajectories; therefore, the absence of short-term utilization reductions should not be interpreted as evidence against longer-term system effects. Fourth, the cost–utility analysis used EQ-5D utilities at baseline and follow-up only, such that QALYs were estimated by linear interpolation; more frequent measurement could capture nonlinear changes and refine incremental benefit estimates. Fourth, the direct medical cost component of the economic analysis is limited to patient-reported out-of-pocket expenditures; insurance reimbursement data from the Urban Employee Basic Medical Insurance and Urban and Rural Resident Basic Medical Insurance funds were not collected and are therefore absent from the cost accounting. This means that total direct medical costs are underestimated in absolute terms for both groups, and the cost differential between groups reflects only the patient-borne financial burden rather than the full resource cost to the healthcare system. Future evaluations should incorporate linked claims data to provide a complete payer or societal cost perspective. Finally, the single-center urban community health-center setting represents an important limitation for generalizability. The study catchment area was exclusively urban, and no rural residents were enrolled. This is a substantive constraint on nationwide generalizability, given that rural China is characterized by markedly lower primary care physician-to-population ratios, weaker family-doctor contracting coverage, higher rates of undiagnosed and uncontrolled chronic disease, and substantially different healthcare utilization patterns compared with urban settings. Residents in rural or peri-urban areas also face distinct financial barriers, including lower insurance reimbursement ceilings under the New Cooperative Medical Scheme and greater reliance on informal caregiving. Consequently, both the clinical effectiveness and economic dominance of the program demonstrated in this urban cohort may not transfer directly to rural contexts, and the cost savings driven in part by reduced productivity loss are particularly sensitive to local wage structures. Multi-center replication in rural county-level facilities and township health centers is a research priority before nationwide scale-up recommendations can be made with confidence. Nevertheless, the favorable dominance observed here, together with the robustness of cost-effectiveness under a 30% higher program-cost scenario, suggests that the economic conclusions are unlikely to be reversed even under less efficient urban implementation conditions. Furthermore, a key limitation is the sole reliance on patient-reported primary outcomes. Because objective clinical indicators (e.g., blood pressure, HbA1c) were not systematically available for formal analysis, the intervention’s effectiveness rests on patient-reported data. Despite validation, this provides a lower level of evidence than objective biomarkers and warrants caution when interpreting clinical effects.

## 5 Conclusion

This prospective cohort study demonstrates that a standardized, primary care-led chronic disease management program significantly improves clinical disease control and health-related quality of life among Chinese adults with noncommunicable diseases. By targeting behavioral mechanisms—specifically medication adherence and self-monitoring—the program achieved superior health outcomes without increasing total societal costs. The economic evaluation identifies the intervention as a dominant strategy generating net cost savings through reduced productivity losses while protecting patients from financial toxicity and cost-related care avoidance. These findings challenge the hospital-centric paradigm of chronic disease care. To translate this evidence into policy, the National Essential Public Health Services Program should mandate structured behavioral follow-up and telehealth monitoring within all hypertension and diabetes family-doctor contracts; performance-based reimbursement should reward adherence outcomes over visit volume; and enrollment resources should prioritize multimorbid and working-age patients who demonstrate the greatest benefit. Rural pilot implementation with rigorous evaluation must precede nationwide scale-up. Sustained investment in community-based management is essential to achieving the equity and efficiency goals of Healthy China 2030.

## Data Availability

The raw data supporting the conclusions of this article will be made available by the authors, without undue reservation.
