# Supplementary material for: Health outcomes, financial protection, and cost-effectiveness of community chronic disease management in China: a cohort study
Source: Front Public Health. 2026 Mar 30;14:1750953. doi: 10.3389/fpubh.2026.1750953 (PMC13071031; doi:10.3389/fpubh.2026.1750953)
Supplement: Supplementary file 1 [file Table_1.DOCX]

| **Supplementary Table S1. Complete Multivariable Model Results for Primary Health Outcomes** | | | | |
| --- | --- | --- | --- | --- |
| **Variable** | **Good Disease Control** | **P Value** | **EQ-5D Utility Score** | **P Value** |
|  | **aOR (95% CI)** |  | **β (95% CI)** |  |
| **Program enrollment (ref: Not enrolled)** | 1.34 (1.05-1.71) | 0.02 | 0.019 (0.005-0.033) | 0.009 |
| **Age (per 10 years)** | 0.92 (0.81-1.05) | 0.21 | -0.008 (-0.015 to -0.001) | .02 |
| **Female sex (ref: Male)** | 1.08 (0.85-1.36) | 0.54 | 0.002 (-0.011-0.016) | 0.75 |
| **Education (ref: No schooling)** |  |  |  |  |
| Primary | 1.12 (0.64-1.97) | 0.68 | 0.015 (-0.018-0.048) | 0.37 |
| Junior middle | 1.18 (0.69-2.02) | 0.55 | 0.018 (-0.014-0.050) | 0.27 |
| Senior high | 1.25 (0.71-2.21) | 0.44 | 0.021 (-0.012-0.054) | 0.21 |
| College/University | 1.31 (0.72-2.38) | 0.37 | 0.025 (-0.009-0.059) | 0.15 |
| Postgraduate | 1.45 (0.61-3.46) | 0.4 | 0.032 (-0.015-0.079) | 0.18 |
| **Household income (ref: <2000 CNY)** |  |  |  |  |
| 2000-4999 CNY | 1.15 (0.78-1.71) | 0.48 | 0.008 (-0.017-0.033) | 0.53 |
| 5000-7999 CNY | 1.22 (0.80-1.86) | 0.36 | 0.012 (-0.015-0.039) | 0.38 |
| 8000-11999 CNY | 1.28 (0.81-2.03) | 0.29 | 0.018 (-0.012-0.048) | 0.24 |
| ≥12000 CNY | 1.35 (0.83-2.19) | 0.23 | 0.022 (-0.009-0.053) | 0.17 |
| Prefer not to answer | 1.08 (0.63-1.86) | 0.78 | 0.005 (-0.025-0.035) | 0.74 |
| **Insurance type (ref: No insurance)** |  |  |  |  |
| UEBMI | 1.42 (0.83-2.44) | 0.2 | 0.028 (-0.005-0.061) | 0.1 |
| URRBMI/NCMS | 1.38 (0.82-2.33) | 0.23 | 0.025 (-0.007-0.057) | 0.12 |
| Private only | 1.25 (0.68-2.30) | 0.47 | 0.021 (-0.016-0.058) | 0.27 |
| Other social insurance | 1.31 (0.74-2.32) | 0.35 | 0.019 (-0.016-0.054) | 0.29 |
| **Primary disease (ref: Hypertension)** |  |  |  |  |
| Type 2 diabetes | 0.87 (0.66-1.14) | 0.31 | -0.018 (-0.035 to -0.001) | .04 |
| Coronary heart disease | 0.74 (0.50-1.10) | 0.13 | -0.035 (-0.058 to -0.012) | .003 |
| COPD | 0.65 (0.38-1.11) | 0.11 | -0.042 (-0.074 to -0.010) | .01 |
| Other | 0.88 (0.48-1.60) | 0.67 | -0.015 (-0.055-0.025) | 0.46 |
| **Years since diagnosis (per year)** | 0.99 (0.97-1.02) | 0.59 | -0.001 (-0.003-0.001) | 0.32 |
| **Number of chronic conditions** | 0.88 (0.78-0.99) | 0.04 | -0.012 (-0.020 to -0.004) | .003 |
| **Serious complications (ref: No)** | 0.82 (0.63-1.08) | 0.15 | -0.021 (-0.038 to -0.004) | .01 |
| **PHQ-2 score (per point)** | 0.91 (0.83-1.00) | 0.05 | -0.008 (-0.013 to -0.003) | .002 |
| **Model performance** |  |  |  |  |
| C-statistic/R-squared | 0.68 |  | 0.15 |  |
| Hosmer-Lemeshow p-value | 0.42 |  | N/A |  |
| AIC | 1723.4 |  | -2845.7 |  |
| *aOR indicates adjusted odds ratio; AIC, Akaike Information Criterion; CI, confidence interval; CNY, Chinese yuan; COPD, chronic obstructive pulmonary disease; EQ-5D, EuroQol 5-dimension; NCMS, New Cooperative Medical Scheme; PHQ-2, Patient Health Questionnaire-2; UEBMI, Urban Employee Basic Medical Insurance; URRBMI, Urban and Rural Resident Basic Medical Insurance. Logistic regression model for good disease control includes 1345 observations with complete data. Linear regression model for EQ-5D utility includes 1345 observations. Both models use robust standard errors clustered by health center registration.* | | | | |

| **Supplementary Table S2. Detailed Annual Cost Components by Program Enrollment Status** | | | | |
| --- | --- | --- | --- | --- |
| **Cost Component** | **Program Enrolled** | **Not Enrolled** | **Incremental Difference** | **P Value** |
|  | **(n=882)** | **(n=463)** | **(95% CI), CNY** |  |
|  | **Mean (95% CI), CNY** | **Mean (95% CI), CNY** |  |  |
| **Direct medical costs** |  |  |  |  |
| Program intervention costs | 1,254 (1,198-1,310) | 0 (0-0) | 1,254 (1,198-1,310) | <.001 |
| Primary care visits | 1,486 (1,389-1,583) | 1,234 (1,125-1,343) | 252 (98-406) | 0.001 |
| Specialist visits | 892 (781-1,003) | 1,156 (1,012-1,300) | -264 (-457 to -71) | .007 |
| Hospital admissions | 2,134 (1,834-2,434) | 2,576 (2,189-2,963) | -442 (-967-83) | 0.1 |
| Emergency department visits | 186 (154-218) | 234 (189-279) | -48 (-101-5) | 0.08 |
| Diagnostic tests | 324 (298-350) | 298 (267-329) | 26 (-12-64) | 0.18 |
| Medications | 1,456 (1,378-1,534) | 1,523 (1,432-1,614) | -67 (-189-55) | 0.28 |
| **Subtotal: Direct medical costs** | 7,732 (7,314-8,150) | 7,021 (6,524-7,518) | 711 (67-1,355) | 0.03 |
| **Direct non-medical costs** |  |  |  |  |
| Transportation | 156 (143-169) | 134 (118-150) | 22 (2-42) | 0.03 |
| Accommodation (for distant patients) | 67 (52-82) | 89 (68-110) | -22 (-48-4) | 0.09 |
| Special diet/supplements | 234 (218-250) | 267 (245-289) | -33 (-62 to -4) | .02 |
| **Subtotal: Direct non-medical costs** | 457 (431-483) | 490 (455-525) | -33 (-78-12) | 0.15 |
| **Indirect costs (productivity losses)** |  |  |  |  |
| Patient time off work | 298 (267-329) | 412 (367-457) | -114 (-175 to -53) | <.001 |
| Family caregiver time | 155 (138-172) | 235 (209-261) | -80 (-113 to -47) | <.001 |
| **Subtotal: Indirect costs** | 453 (419-487) | 647 (598-696) | -194 (-257 to -131) | <.001 |
| **Total annual costs (societal perspective)** | 8,642 (8,124-9,160) | 9,158 (8,461-9,855) | -516 (-1,389 to 357) | .25 |
| **Alternative perspectives** |  |  |  |  |
| Healthcare system perspective only | 6,842 (6,324-7,360) | 7,458 (6,761-8,155) | -616 (-1,489 to 257) | .16 |
| Patient out-of-pocket only | 1,234 (1,145-1,323) | 1,456 (1,334-1,578) | -222 (-371 to -73) | .003 |
| **Cost per participant by program intensity** |  |  |  |  |
| Low intensity (1-2 visits) | 8,156 (7,423-8,889) | N/A | -1,002 (-2,032 to 28) | .06 |
| Medium intensity (3-4 visits) | 8,534 (7,945-9,123) | N/A | -624 (-1,513 to 265) | .17 |
| High intensity (≥5 visits) | 9,123 (8,456-9,790) | N/A | -35 (-1,123 to 1,053) | .95 |
| **Sensitivity analyses** |  |  |  |  |
| 30% higher program costs | 9,018 (8,500-9,536) | 9,158 (8,461-9,855) | -140 (-1,013 to 733) | .75 |
| 50% lower productivity costs | 8,416 (7,898-8,934) | 8,835 (8,138-9,532) | -419 (-1,292 to 454) | .35 |
| Excluding informal care costs | 8,487 (7,969-9,005) | 8,923 (8,226-9,620) | -436 (-1,309 to 437) | .33 |
| *CI indicates confidence interval; CNY, Chinese yuan. All costs adjusted to 2024 CNY using medical care price indices. Program intervention costs include personnel time, facility overhead, materials, and telehealth platform fees. Productivity costs calculated using human capital approach with age- and sex-specific wage rates. Healthcare system perspective excludes patient out-of-pocket costs, transportation, and productivity losses. Confidence intervals calculated using 1000 bootstrap replicates. P values from generalized linear models with gamma distribution and log link, adjusted for baseline characteristics.* | | | | |

| **Supplementary Table S3. Missing Data Patterns and Sensitivity Analysis** | | | | |
| --- | --- | --- | --- | --- |
| **Variable** | **Complete Data** | **Missing Data** | **Missing Pattern** | **Imputation Method** |
|  | **n (%)** | **n (%)** |  |  |
| **Baseline characteristics** |  |  |  |  |
| Age | 1345 (100.0) | 0 (0.0) | Complete | None required |
| Sex | 1345 (100.0) | 0 (0.0) | Complete | None required |
| Education | 1345 (100.0) | 0 (0.0) | Complete | None required |
| Household income | 1262 (93.8) | 83 (6.2) | Prefer not to answer | Separate category |
| Insurance type | 1345 (100.0) | 0 (0.0) | Complete | None required |
| **Clinical variables** |  |  |  |  |
| Primary disease | 1345 (100.0) | 0 (0.0) | Complete | None required |
| Disease control status | 1345 (100.0) | 0 (0.0) | Complete | None required |
| EQ-5D utility | 1345 (100.0) | 0 (0.0) | Complete | None required |
| **Psychosocial variables** |  |  |  |  |
| PHQ-2 total score | 1345 (100.0) | 0 (0.0) | Complete | None required |
| Family support | 1232 (91.6) | 113 (8.4) | Random | Multiple imputation |
| Friend support | 1074 (79.9) | 271 (20.1) | Random | Multiple imputation |
| Health literacy | 1298 (96.5) | 47 (3.5) | Random | Multiple imputation |
| **Cost variables** |  |  |  |  |
| Healthcare utilization | 1321 (98.2) | 24 (1.8) | Random | Multiple imputation |
| Out-of-pocket costs | 1289 (95.8) | 56 (4.2) | Random | Multiple imputation |
| Productivity losses | 1278 (95.0) | 67 (5.0) | Random | Multiple imputation |
| **Sensitivity analyses** |  |  |  |  |
| Complete case analysis (n=1156) | Program effect on disease control: aOR 1.31 (1.01-1.69), p=.04 |  | | |
| Multiple imputation (n=1345) | Program effect on disease control: aOR 1.34 (1.05-1.71), p=.02 |  | | |
| Best case scenario | Program effect on disease control: aOR 1.42 (1.12-1.80), p=.004 |  | | |
| Worst case scenario | Program effect on disease control: aOR 1.23 (0.97-1.56), p=.09 |  | | |
| *aOR indicates adjusted odds ratio; PHQ-2, Patient Health Questionnaire-2. Multiple imputation performed using chained equations with 20 imputations. Best case scenario assumes missing outcomes favor program enrollment. Worst case scenario assumes missing outcomes favor control group. Missing data patterns assessed using Little's MCAR test (p=.34, suggesting missing completely at random). All sensitivity analyses show consistent direction of effect for primary outcomes.* | | | | |
